# Collections of Pus in the Cavities of the Heart
*Journ. Hebdomad. No. 25.


**Published:** 1829-10-01

**Authors:** 


### HOPITAL DES ENFANS.

### COLLECTIONS OF PUS IN THE CAVITIES OF THE HEART.*

How much has been done in the investigation of diseases of the heart since the times immediately preceding that of Corvisart, we need not stop to inform our readers. That celebrated physician, and the immortal Laennec, have conferred a degree of scientific accuracy on our diagnostic and pathological acquaintance with the morbid alterations of the organ, of which they appeared to be scarcely susceptible. In the ruder days of medicine the heart was always the chosen spot for monstrous diseases and incredible appearances, most of which have vanished before the subdued but steady light of modern discovery. There are still, however, affections of the organ which we do not understand, and are therefore apt to view with scepticism, if not with absolute distrust; of such a nature, are two cases reported from the Hôpital des Enfans, by M. Maréchal, interne of that institution.

CASE 1. *Paraplegia—Inflammation of the Lungs—Hypertrophy with Dilatation of the Heart—Malformation of the Right Ventricle—Purulent Depôt in the Left.*

F. Clementine, 14 years of age, of good constitution, but who had not yet menstruated, entered the hospital on the 31st of January, 1828, with almost perfect paraplegia. Caustic issues to the loins and warm vapour douches proving unsuccessful, M. Guersent administered the nux vomica in the form of lavement. The remedy proved of considerable service, and the dose was raised to five grains and a half, when symptoms of gastro-entérite supervened, and its use was abandoned. The symptoms in question subsided under moderate antiphlogistic regimen, but the paraplegia gradually returned, and the patient was soon in the same state as when she was first received in the hospital.

About the month of May, she was seized again with pain in the abdomen, fever, head-ache, nausea, and diarrhœa, together with cough and much mucous expectoration. Local bleedings were employed without any great effect, but the symptoms were not productive of alarm until after a violent mental emotion, when they assumed an extremely dangerous aspect. The pulse became rapid and extremely weak, the expression of the countenance anxious and altered, the cough was harrassing, painful, and attended with a continual sense of suffocation—the nausea was converted into frequent vomiting of dark-coloured matters—the diarrhœa gave way to obstinate constipation with violent pain, sometimes in the epigastric, sometimes in the hypogastric region—the surface of the body was cold—the left foot red, and extremely painful. The application of external warmth, with derivatives and narcotics, was resorted to in vain, and the symptoms continued unabated for two days, when a copious alvine evacuation procured a temporary calm. They quickly, however, returned with augmented violence, especially the coldness of the lower extremities and imminent sense of suffocation. In a paroxysm of the latter the patient expired at 7, a.m. of the 1st of June.

*Dissection.* The contents of the cranium were perfectly sound. The spine presented a lateral curvature, but exercised no compression on the medulla, which was free from disease, as were also its membranes. The bronchial glands were large, and the principal divisions of the bronchi were deeply reddened, and filled with a frothy bloody fluid, resembling the expectoration during the two last days of the patient's life. On the left side of the chest were some old adhesions of the pleuræ, and the apex of that lung presented a tuberculous excavation, surrounded by a hardened mass, in part hepatized, and in part infiltrated with tubercles. The lower lobe was much gorged, and in its centre were several nuclei of hepatization. The right lung was also gorged with blood, and crepitated little throughout.

The heart was very large, measuring

* Journ. Hebdomad. No. 25.

five inches in length from base to apex, and four inches at its greatest breadth. This enlargement depended not only on dilatation of the cavities, but thickening of the parietes; in other words, on hypertrophy with dilatation. The left ventricle particularly shewed this alteration, being more than doubled in the size of its cavity, and presenting columnæ carneæ as large as the little finger of an adult. The hypertrophy of the walls of the ventricle was greatest at the base, the apex being thin and weak. The inferior half of the ventricle was filled by a round, white, fluctuating mass, containing in its centre a greyish-red liquid, of the consistence of cream, around a sort of nucleus marked with whitish streaks. The mass in question was soft, lacerable, and adhering slightly to the columnæ carneæ, between which it sent prolongations. The rest of the ventricular cavity contained several loose and dark coagula. The aortic opening was so narrow as scarcely to receive the extremity of the fore-finger, and the artery itself was of very small calibre. The left auricle was diminutive. The right ventricle was only dilated at its upper part, which was filled by black and soft coagula; across its middle was a transverse band, partitioning its cavity into two; this septum, which does not appear to have been a complete one, was three lines in breadth, and its extremities were continuous with the columnæ carneæ. The right auricle was much distended and filled with black coagulated blood.

The mucous membrane of the stomach and intestines was reddened throughout its whole extent. Small rounded tubercles, the size of peas, were deposited in the cortical substance of the kidneys.

CASE 2. *Hepatization of one Lung—Pus in the Cavities of the Heart—Ulcer in the Intestines, &c.*

Marguérite Menjaud, ten years of age, of weak constitution, was admitted into the Hôpital des Enfans on the 28th of August, 1828, with stupor, prostration, and other symptoms of severe fever. She had only been ill some days before her admission, and at the end of a month the parents removed her from the house, although she was far from convalescent. In another month, however, they brought her back, stating that the child had suffered from cough and diarrhœa, which had been much worse for the last four days, during which there had been vomiting and palpitations at the heart. In the course of a week, which elapsed between the patient's re-admission, and her death, the following symptoms were observed. Pulse not frequent, but extremely small—pulsations of the heart very violent, unaccompanied with any particular *bruit*, but perceptible in the epigastric region, and heard over the whole extent of the thorax—cough constant—sputa viscid, glutinous, and yellow—dull sound on percussion behind and on the right side—absence of respiration, and "souffle tubaire" in the same points—slight tremor of the voice—decubitus on the right side—nausea—diarrhœa—and pain over the whole extent of the abdomen. Such were the symptoms observed in the short space of time that elapsed before the little patient died.

*Dissection.* The substance of the brain was extremely firm. The bronchial glands on the right side were softened, and infiltrated in part with tuberculous matter; on the left they were merely enlarged and red. The two upper lobes of the right lung were crepitant throughout, and contained some grey, semi-transparent granules. The inferior lobe was completely hepatized, and contained above, some small tubercular masses: and below, a tubercle the size of a pigeon's egg, and softened in its centre. The right pleural cavity contained some greenish, rather turbid liquid, and the pleura pulmonalis was covered by a soft false membrane. The right side of the chest was unaffected.

The volume of the heart was at least doubled from the equal dilatation of the two ventricles, without appreciable augmentation of the thickness of their walls. Both ventricles were filled with black clots, "beneath which were seen three purulent collections. One was on the left side, lying on the inter-ventricular septum at about its middle; the others were on the right. On both sides they presented the same characters, being about the volume of a small nut, white externally, in contact with the coagula and columnæ carneæ, and sending prolongations into the intervals between the last. Each pouch in the interior contained a fluid, having the consistence of cream, of a dirty yellow colour, and entirely escaping on wounding the pouch. The latter was about a third of a line in thickness, of dull white colour, slightly rugous, and easily broken.

The mesenteric glands had small tuberculous points in their centre. The mucous membrane of the stomach was softened and wasted in parts. Above the ileo-cæcal valve, was a lenticular ulceration of the small intestine. The mucous membrane of the valve, the cæcum, and the ascending colon, were uniformly red. The spleen presented in its texture a number of solid tubercular deposits. The same were discovered in the liver, containing in their centre a greenish liquid, resembling in all respects the bile.

These cases may afford matter for some consideration and more dispute. Waving on this occasion all other points, we must dwell for a moment on what was discovered in the heart. Abscess in the centre of that organ, though a somewhat startling kind of announcement, has yet been alluded to by authors. Mr. Wardrop, in his edition of Baillie's Morbid Anatomy, declares that he has seen "a preparation of a polypus in the left ventricle, in the centre of which was a distinct abscess;" and Mr. J. Stewart relates a case in the Edinburgh Medical and Surgical Journal for 1817, in which highly vascular polypi were discovered in the right auricle, and either ventricle. We think, then, that taking these facts, and especially that of Mr. Wardrop, in connexion with the two now reported from the Hôpital des Enfans, little doubt can remain, that either purulent matter, or something so like it as completely to deceive the best informed men, has been found in coagula or polypi lodged in the cavities of the heart. To this conclusion we must either yield our undivided assent, or believe that Mr. Wardrop, and the medical officers of the Hôpital des Enfans, have been themselves misled, or wilfully misled their brethren. The existence of pus is, however, the least of the difficulty; its mode of formation is the rub. Are we to suppose that it is produced in the centre of a polypus, that is to say, in a clot of unorganized matter, by the same sort of process as in organized parts of the living body; in other words, that it is the product of inflammation? Our reason, or it may be our prejudice, forbids the supposition. There were no other marks of inflammation present, no lymph, no injection of vessels, and the symptoms during life differed so materially in the only two cases of which we have given an account, as to disprove, to our minds, the idea of its existence.

Whoever has examined many polypi of the heart must have noticed that they frequently vary in their consistence. The central part is occasionally softer, and yellower or paler in colour than the external, and that to a very considerable degree. It may readily be imagined that such a change may proceed even farther, and instead of mere softening, that actual pus, or something very like it, may be formed, independently of all cognizable inflammation, or of any thing akin to such a process. Pus is a product of the blood, and we see no good reason against its formation, under peculiar circumstances, from its matrix, by other means than by inflammatory action. At all events, it is not only more safe, but more philosophical, to conclude that the purulent fluid found in these cases was the result of some peculiar decomposition or change in the clot itself, than of inflammation resembling that in other parts. Analogy is against the latter supposition, probability in favour of the former.

The reporter of the present cases believes that the pus was formed during life. Without ripping up the ancient disputes respecting the ante-mortem or post-mortem origin of polypi, we are certainly inclined to differ from the reporter. If the purulent matter was produced before death, as he supposes, then the coagula must have been so too, which is contrary to the opinion at present universally held upon the subject.

